# Benchmarking of AlphaFold2 accuracy self-estimates as indicators of empirical model quality and ranking: a comparison with independent model quality assessment programmes

**DOI:** 10.1093/bioinformatics/btae491

**Published:** 2024-08-08

**Authors:** Nicholas S Edmunds, Ahmet G Genc, Liam J McGuffin

**Affiliations:** School of Biological Sciences, University of Reading, Whiteknights, Reading, RG6 6EX, United Kingdom; School of Biological Sciences, University of Reading, Whiteknights, Reading, RG6 6EX, United Kingdom; School of Biological Sciences, University of Reading, Whiteknights, Reading, RG6 6EX, United Kingdom

## Abstract

**Motivation:**

Despite an increase in protein modelling accuracy following the development of AlphaFold2, there remains an accuracy gap between predicted and observed model quality assessment (MQA) scores. In CASP15, variations in AlphaFold2 model accuracy prediction were noticed for quaternary models of very similar observed quality. In this study, we compare plDDT and pTM to their observed counterparts the local distance difference test (lDDT) and TM-score for both tertiary and quaternary models to examine whether reliability is retained across the scoring range under normal modelling conditions and in situations where AlphaFold2 functionality is customized. We also explore plDDT and pTM ranking accuracy in comparison with the published independent MQA programmes ModFOLD9 and ModFOLDdock.

**Results:**

plDDT was found to be an accurate descriptor of tertiary model quality compared to observed lDDT-Cα scores (Pearson *r* = 0.97), and achieved a ranking agreement true positive rate (TPR) of 0.34 with observed scores, which ModFOLD9 could not improve. However, quaternary structure accuracy was reduced (plDDT *r* = 0.67, pTM *r* = 0.70) and significant overprediction was seen with both scores for some lower quality models. Additionally, ModFOLDdock was able to improve upon AF2-Multimer model ranking compared to TM-score (TPR 0.34) and oligo-lDDT score (TPR 0.43). Finally, evidence is presented for increased variability in plDDT and pTM when using custom template recycling, which is more pronounced for quaternary structures.

**Availability and implementation:**

The ModFOLD9 and ModFOLDdock quality assessment servers are available at https://www.reading.ac.uk/bioinf/ModFOLD/ and https://www.reading.ac.uk/bioinf/ModFOLDdock/, respectively. A docker image is available at https://hub.docker.com/r/mcguffin/multifold.

## 1 Introduction

Protein modelling software routinely provides accuracy self-estimate (ASE) scores to accompany the models constructed (Varadi *et al.* 2022), and while competitive modellers are mainly concerned with correlations and statistical measures of significance across large datasets, to the general biologist, the accuracy and usefulness of a single predicted score for one or only a few models may be more important. Since the success of AlphaFold2 (AF2) (Jumper *et al.* 2021) at CASP14, the methodology behind the AlphaFold process has been the subject of many research articles (Evans *et al.* 2022) and the AF2 ASE scores, plDDT and pTM, have become often quoted useful indicators of tertiary model quality (Takei and Ishida 2022). However, it is unclear whether the reliability of these scores in describing tertiary model quality extends to quaternary structure modelling or if there are occasions when the accuracy of either of these two scores should be questioned.

### 1.1 The three AF2 ASE scores PAE, plDDT, and pTM

Although AF2 produces three ASE scores, this study concentrated on plDDT and pTM only, and there are a number of reasons for this. Firstly, PAE (predicted alignment error) is not automatically normalized by AF2 into a single overall score, making plDDT and pTM the most often quoted AF2 confidence metrics for both tertiary and quaternary structure models. Secondly, the default ranking of AF2 models is by plDDT and AF2-Multimer models is by pTM (Evans *et al.* 2022) (see Supplementary Section S1.1) and, lastly, both scores have observed counterparts in the local distance difference test (lDDT) and TM-score against which they can be directly measured.

plDDT is based on the lDDT-Cα version (Tunyasuvunakool *et al.* 2021) of lDDT (Mariani *et al.* 2013) which estimates confidence by comparing distances in the local arrangement of amino acid Cα atoms. It is useful for assessing the local accuracy of domains as it will not penalize incorrect relative domain orientation if there is a good match between inter-atomic distances. plDDT is output as both a per-residue score in the B-factor column of an AF2 model coordinates file and also as a global per-model score in the modelling log file. It has a range of 0–100 (lDDT values are also quoted in the 0–1 range), and values ≥90 equate to high confidence, those between 90 and 70 as confident, from 70 to 50 as low confidence and <50 as very low confidence with a tendency for disorder (Varadi *et al.* 2022).

pTM is based on the topological similarity score TM-score (Zhang and Skolnick 2004) and is calculated from the PAE matrix (Wallner 2023). In later AlphaFold2 versions, this is also output in the modelling log file and has a range of 0–1. No published confidence boundaries could be found for pTM but a TM-score of 1.0 represents a perfect match between a model and its native structure, a score greater than 0.5 represents the same globular fold, and scores below 0.17 are associated with unrelated proteins (Zhang and Skolnick 2004). Jumper *et al.* (2021) described a pTM versus TM-score relationship as TM-score = 0.98 × pTM + 0.07 and so it may be appropriate to artificially construct pTM confidence boundaries on this basis.

### 1.2 Documented descriptions of AlphaFold2 predicted scores

One of the strengths of the AF2 algorithm has been described as its ability to recognize low-accuracy areas of models (Shao *et al.* 2022) and apply ASE scores appropriately. Linear relationships between ASE and observed scores have also been described, suggesting that, despite a tendency for some minor over-prediction with plDDT (Jumper *et al.* 2021, Tunyasuvunakool *et al.* 2021), both scores are consistently applied across the scoring range. However, at CASP15, it was noticed that there was a variability in these scores, particularly for multimer models of similar quality. One group (Wallner 2023) reported that up to one-third of models with a pTM score > 0.8 had the wrong domain orientation and our own modelling experiences revealed an increase in plDDT of up to 40 points during refinement by recycling, which would suggest an over-estimate of model quality improvement.

### 1.3 Wider uses of AlphaFold2 rely on accurately predicted quality

AF2 has been used in a DeepMind-EMBL collaboration to create the AlphaFold Protein Structure Database (Tunyasuvunakool *et al.* 2021), a community resource of predicted protein structures which remain unsolved by experimental methods. Although, for now, the database is limited to tertiary structures, it might, nevertheless, be prudent to examine whether AF2’s confidence metrics can be relied upon to rate and rank models accurately across the whole quality range. Further to this, at least three published works describe using ColabFold (Mirdita *et al.* 2022) to input models as custom templates. One group (Terwilliger *et al.* 2022) described inputting electron density maps from experimental data, another (Adiyaman *et al.* 2023) described a model refinement procedure using custom template recycling in which full 3D model files were submitted via the custom template facility available in the ColabFold versions to be recycled through the system with the five output models quality-assessed and ranked by plDDT and pTM is the usual way. A third group (Roney and Ovchinnikov 2022) described a similar method for using AF2 as a quality assessment (QA) tool. Clearly, reliance is being placed on plDDT and pTM scores, but it appears that for multimeric models and those where custom processing is used, there may be occasions when published accuracy levels are not maintained.

### 1.4 Objectives

This study used blind modelling and EMA data from CASP15 to assess the performance of plDDT and pTM in both tertiary structure (monomer) and quaternary structure (multimer) model populations in comparison to their observed lDDT and TM-score counterparts. Model populations were generated with and without custom template recycling to evaluate whether a difference in predictive performance could be detected with this single variable. In addition, plDDT and pTM were compared to scores generated by the independent model quality assessment (MQA) programmes ModFOLD9 (for tertiary structure models) (McGuffin and Alharbi 2024) and ModFOLDdock (for quaternary structure models) (McGuffin *et al.* 2023). See Supplementary Section S2.7 for descriptions of both of these scores.

Two hypotheses were formulated to test the AF2 scores’ accuracy at describing models. The first was intended to test for overprediction of predicted scores versus their observed global counterparts:

*H0. There is no difference in magnitude between the AF2 predicted and equivalent observed scores*

*H1. The magnitude of the AF2 predicted scores is greater than the equivalent observed scores.*

The second was intended to test model ranking agreement with observed scores.

*H0. There is no association between the AF2 predicted and observed score ranking categories.*

*H1. There is an association between the AF2 predicted and observed score ranking categories.*

A third hypothesis was formulated to assess the comparative ranking performance between ModFOLD9 and AF2 scores for tertiary structure models and ModFOLDdock and AF2-Multimer scores for quaternary structures (blind ModFOLD9 predictions were run in house prior to the release of the CASP15 experimental structures).

*H0. There is no difference between the independent QA and AF2 rankings as measured by the association between model rank categories.*

*H1. Independent QA and observed score model ranks are more closely associated than AF2 and observed score model ranks.*

Finally, we examined the effect of custom template recycling on the accuracy of the AF2 and AF2-Multimer predicted scores. These results are described in Supplementary Section S3.4. Hypothesis four stated:

*H0. There is no difference between AF2 regular modelling and custom template modelling predicted scores, when compared to equivalent observed scores.*

*H1. AF2 predicted scores following custom template modelling show greater variation than scores from regular modelling, when compared to equivalent observed scores*.

## 2 Materials and methods

### 2.1 Selection of models to test the hypotheses

Four individual datasets were used for this study (Table 1). Population A (CASP15 monomers) comprised the McGuffin group’s tertiary structure submissions for CASP15. Population B (CASP15 multimers) was composed of both the McGuffin group’s (MultiFOLD, group 462) and the ColabFold group’s (group 446) multimer submissions for CASP15 (group 446 submissions are publicly available from https://casp15.colabfold.com/). Population C (recycled monomers) is a superset (20 targets) of the models used in the custom-template recycling experiment described in our previous paper (Adiyaman *et al.* 2023). The original recycled model population had been fixed at 16 CASP14 targets forming a common FM-target subset with the ReFOLD4 molecular dynamics analysis, which was included in the previous paper. The emphasis for this experiment had shifted from measuring model improvement to global model quality and so four additional FM/TBM targets, for which scores had already been collected, were included to increase the model population without significantly altering model difficulty. Population D (recycled multimers) is the same multimer population used in the custom-template recycling experiment described in our previous paper. Exact processing details of each dataset including the CASP targets used can be found in Supplementary Sections S2.2–S2.5 but a short overview is given below.

**Table 1.** A summary of the model populations used in the study.

Population	Source and model software	Stoichiometry and modelling
A1	CASP15, MultiFOLD R1	Monomer, regular modelling
A2	CASP15, MultiFOLD R2	Monomer, custom recycling
B1	CASP15, ColabFold	Multimer, regular modelling
B2	CASP15, MultiFOLD	Multimer, custom recycling
C	CASP14, AF2 and non-AF2	Monomer, custom recycling
D	CASP14, top 5 groups	Multimer, custom recycling

Custom recycling means that custom template recycling is used in the modelling process.

### 2.2 The Population A dataset: CASP15 monomer models

This consisted of all McGuffin group’s blind model submissions for 26 CASP15 regular tertiary structure targets for which ModFOLD9 scores and a reference native structure were available. Two separate modelling rounds were used; round 1 (Population A1) used regular modelling and no refinement process, whereas round 2 (Population A2) included refinement by MultiFOLD custom template recycling. Predicted plDDT and pTM scores were taken directly from the server for all models and predicted ModFOLD9 scores were collected from the original cached datasets used during CASP15. Observed lDDT and TM-scores were generated using the downloadable versions of TM-score (Zhang and Skolnick 2004) and lDDT score (Mariani *et al.* 2013) to compare models for each target with the CASP observed structures. A total of 735 models were analysed consisting of 490 round 1 and 245 round 2 models.

### 2.3 The Population B dataset: CASP15 multimer models

This comprised all blind multimer (assembly) CASP15 model submissions for the MultiFOLD (462) and ColabFold (446) group servers. These two sets were chosen because they were created using the same base ColabFold software (exact versions may differ) but differed by the use of custom template recycling in the MultiFOLD pathway. The rationale was that the ColabFold models could be used to assess AF2-Multimer score overprediction during regular modelling and, that by comparing the ColabFold and MultiFOLD populations, the effect of the additional custom template recycling on predicted scores could be assessed. The ColabFold group multimers are named Population B1 and MultiFOLD group models are named Population B2. Scores for rank 1–5 models were collected for all multimer models for which data were available, resulting in 395 individual models across 41 targets (the ColabFold group submitted no models for three targets making a total of 38). In total the Population B dataset consisted of 395 multimer scores.

### 2.4 The Population C dataset: recycled monomer models

This dataset consisted of custom template recycled AF2 and non-AF2 tertiary models. The AF2 dataset contributed 800 individual scores from 8 sets of scores per model across 5 models per target for 20 targets. Non-AF2 models were selected from the same 20 FM targets for the next five best-ranked groups beneath AlphaFold2 at CASP14. These were Baker (473), Baker-experimental (403), Feig-R2 (480), Zhang (129), and tFold_human (009). To ensure consistency in terms of globular fold similarity, only models with a TM-score ≥0.45 were selected, resulting in 47 individual models with a total of 1880 individual model scores.

### 2.5 The Population D dataset: recycled multimer models

This dataset consisted of custom template recycled multimer models. As the AlphaFold2 group did not submit multimer (assembly) models at CASP14, models for this dataset were selected from the CASP14 top five ranked groups. According to official results tables, these were Baker, Venclovas, Takeda-Shitaka, Seok, and DATE. This dataset contributed a total of 2000 individual scores.

An overall total of 5810 model scores were collected across the whole study. The method for handling multi-contingency table data and ranking by pTM is described in Supplementary Section S2.6.

## 3 Results

Supplementary figures and tables referred to below can be found under Supplementary Section S3.

### 3.1 Hypothesis 1: are AF2-predicted scores higher than the equivalent observed scores?

In order to focus on one independent variable at a time, the question of whether predicted scores are good quality indicators must be answered using only models which have *not* undergone custom template recycling, as this may act as a confounding factor. For monomers, this is Population A1 (round 1 models) and for multimers it is Population B1 (ColabFold multimers). Population A1 will be considered first.

#### 3.1.1 Part 1: monomer data; Population A1 (round 1)

AF2 default monomer ranking is by plDDT and so results will focus on plDDT/lDDT similarity.

Although plDDT scores were found to be elevated compared to the all-atom lDDT scores (see Supplementary Fig. S1), when plDDT scores were compared to lDDT-Cα scores, which represent them more closely (Tunyasuvunakool *et al.* 2021), there was no evidence of plDDT over-prediction, infact the plots in Supplementary Fig. S2 show a slightly lower median score for plDDT values. To formally test this data against Hypothesis 1, a Shapiro test was performed showing that all score distributions were non-normal, followed by a Wilcoxon signed rank test for non-parametric paired data to test significance. Wilcoxon results are shown in Table 2, rows 1–4 and agree that, while a significant difference between predicted and observed values for both lDDT and lDDT-Cα scores was detected by a two-sided test (*P*-values of 2.2 ×10^−16^ for all-atom lDDT and 9.69 × 10^−6^ for lDDT-Cα), a one-sided test showed that plDDT values were actually significantly lower than lDDT-Cα values (*P*-value of 4.81 × 10^−6^). Considering the published works cited above confirming that plDDT is based on lDDT-Cα it would be appropriate to accept the null hypothesis in this case. Therefore, for monomers using regular straight-forward AF2 modelling compared to lDDT-Cα score: ‘There is no increase in magnitude between the AF2 predicted and equivalent observed scores’.

**Table 2.** Wilcoxon test statistics for monomer population A1 (rows 1–4, unshaded) and multimer population B1 (rows 5–8, shaded).^a^

Scores compared	Independence and distribution symmetry	*P*-values
plDDT versus lDDT	Paired; two-sided test	2.2 × 10^−16^
plDDT versus lDDT	Paired; one-sided (plDDT>lDDT)	2.2 × 10^−16^
plDDT versus lDDT-Cα	Paired; two-sided test	9.7 × 10^−6^
plDDT versus lDDT-Cα	Paired; one-sided (plDDT<lDDT-Cα)	4.81 × 10^−6^
plDDT versus oligo-lDDT	Paired; one-sided (plDDT>oligo-lDDT)	2.2 × 10^−16^
pTM versus TM-score	Paired; two-sided	0.038
pTM versus TM-score	Paired; one-sided (pTM>TM-score)	0.980
pTM versus TM-score	Paired; one-sided (pTM<TM-score)	0.019

a *P*-values are calculated at the 95% confidence threshold meaning that values <0.05 are considered significant.

#### 3.1.2 Part 2: multimer data; Population B1 (ColabFold multimers)

For multimers pTM is the default ranking metric, however, plDDT was used in early versions of ColabFold and so both scores are considered.

**Figure 1.**
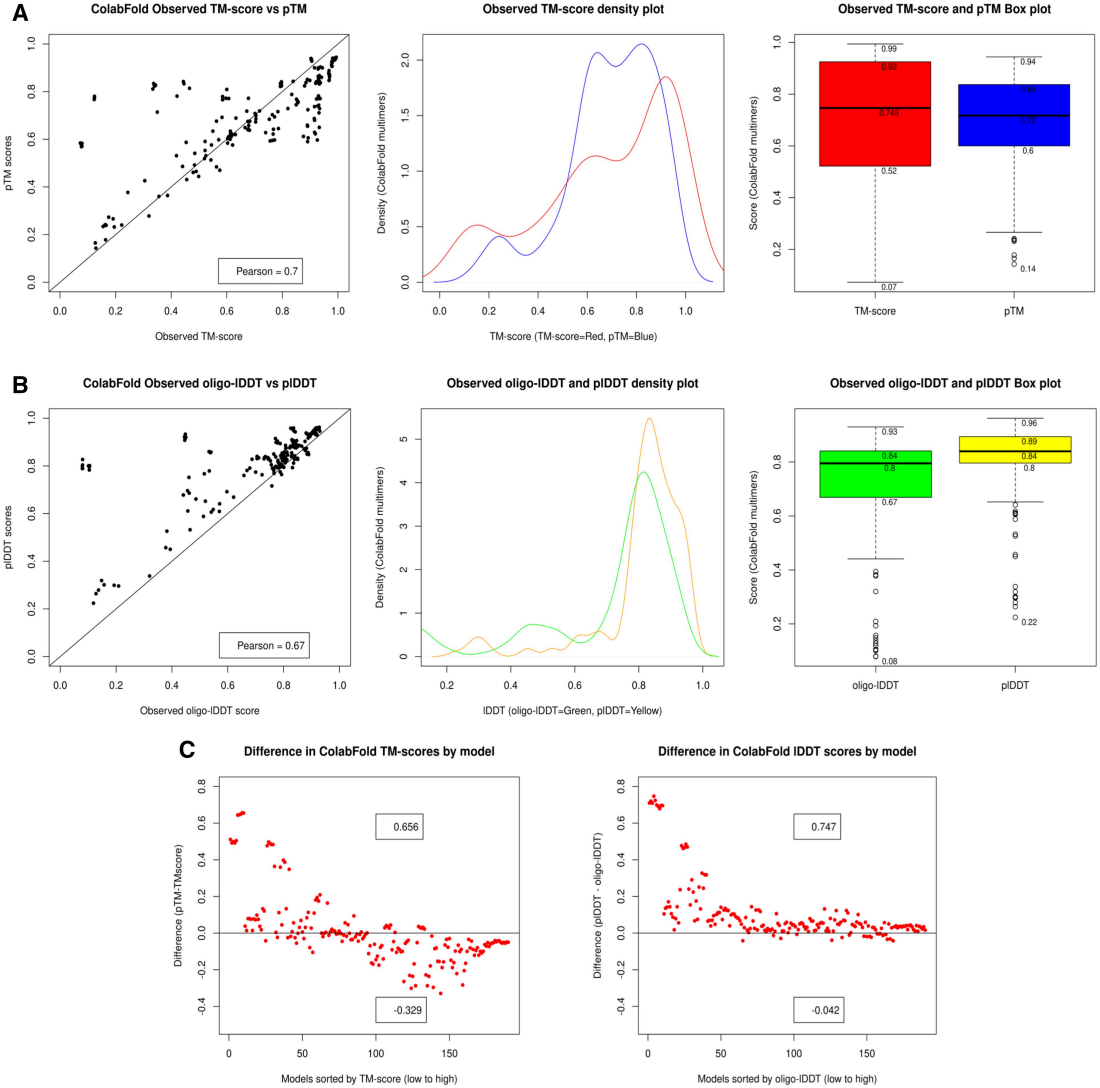
Plots showing predicted versus observed score distributions. (A) A scatter plot (left), density plot (middle) and boxplot (right) showing pTM versus observed TM-score for Population B1 (ColabFold multimers). (B) Similar plots for plDDT versus observed oligo-lDDT also for Population B1 (ColabFold multimers). (C) Differences between pTM and observed TM-score (left) and plDDT and observed oligo-lDDT score (right) for population B1 (ColabFold multimers). The horizontal line at 0.0 represents the observed score and the *x*-axis scale represents models in the population, ordered from low to high observed score. plDDT is rescaled to the 0–1 range for all plots

In Fig. 1A, both the scatter and density plots show an underestimation of pTM score for higher quality multimer models but a relatively large overestimation for some lower-quality models. For Fig. 1B, plDDT is largely overestimated across the quality range which may be partially accounted for by the use of an all-atom observed oligo-lDDT score (see Supplementary Section S2.7). However, as with pTM scores, there is a more pronounced overestimation for some models in the lower quality range. The Shapiro test for normality (distributions were non-normal) and Wilcoxon signed rank test for significance were used in the same way as described for monomer data. As shown in Table 2, row 5 (shaded), a significant difference was detected between predicted plDDT and observed oligo-lDDT scores and that plDDT values were significantly higher than their oligo-lDDT counterparts (*P*-value of 2.2 × 10^−16^). For hypothesis 1, with respect to oligo-lDDT, the alternative hypothesis must therefore be accepted for ColabFold multimers, i.e. ‘The magnitude of the AF2 predicted scores is higher than the equivalent observed scores’.

The data were not so clear for pTM scores. The Wilcoxon tests (Table 2, rows 6–8) showed a marginally significant difference between pTM and TM-score but rather than pTM being the greater of the two (*P*-value of .980), TM-score was, in fact, the greater (*P*-value of .019). To reveal more information about the relationship between pTM and TM-score, a closer investigation into the variation in the two scores was carried out.

The relationships suggested in Fig. 1A and B are more clearly shown by the two plots in Fig. 1C. Both plots show that an overestimation of predicted scores is more likely for lower-quality models, with a maximum difference of +0.65 for pTM and +0.74 for plDDT. Also, the tendency for the underestimation of pTM for higher quality models is more clearly shown, with a maximum difference of −0.32. This explains the unclear Wilcoxon test results for pTM; there is both over and under-estimation occurring which is quality-related and which, to some extent, cancel each other out. While there is an allusion to minor pTM underprediction in the documented linear relationships (Jumper *et al.* 2021), no documentation relating to an overprediction for lower quality models could be found. A similar pattern of underestimation is not seen for plDDT.

For hypothesis 1, with respect to TM-score, the null hypothesis must be accepted for ColabFold multimers due to the unclear Wilcoxon result, i.e. ‘There is no increase in magnitude between the AF2 predicted and equivalent observed scores’. However, a caveat can be added, that, for regular multimer modelling, pTM overprediction was apparent in models of lower observed quality which may have been masked by simultaneous underprediction of higher-quality models.

### 3.2 Hypothesis 2: is AF2 model ranking reliable as measured by association with observed model rank categories?

Again, to answer this question fairly, models which had not undergone custom template recycling were used, i.e. the same Populations A1 (round 1 models) and B1 (ColabFold multimers) models used in 3.1.

For monomer data, results showed strong agreements between observed lDDT-Cα derived ranks and plDDT-predicted ranks. The high level of agreement for rank 1 and rank 5 data (see contingency table A in Supplementary Fig. S3) is supported by results in Table 3 (rows 1–6) showing a mean true positive rate (TPR) of 34.28%, a Fisher’s exact test *P*-value well below the significance level of .05 and a Chi-squared test value of 167.35 with a *P*-value of 2.2 × 10^−16^. These data provide robust evidence that this distribution was unlikely to have occurred by chance and that there is a significant positive relationship between the predicted and observed score ranks.

**Table 3.** Ranking agreement summary statistics for monomer models not subject to custom template recycling.^a^

Row	Test (monomers)	plDDT versus lDDT score	plDDT versus lDDT-Cα
1	Macro-sensitivity (TPR)	0.3204	0.3428
2	Macro-specificity	0.8301	0.8357
3	Macro-precision	0.3204	0.3428
4	Macro-accuracy	0.7281	0.7371
5	Fisher’s exact (*P*-value)	<.001	<.001
6	Chi-squared (*χ*^2^; *P*-value)	128.27; 2.2 × 10^−16^	167.35; 2.2 × 10^−16^

Row	Test (monomers)	MF9 versus lDDT score	MF9 versus lDDT-Cα
7	Macro-sensitivity (TPR)	0.2551	0.2693
8	Macro-specificity	0.8137	0.8173
9	Macro-precision	0.2551	0.2693
10	Macro-accuracy	0.7020	0.7077
11	Fisher’s exact (*P*-value)	<.001	<.001
12	Chi-squared (*χ*^2^; *P*-value)	69.93; 2.5 × 10^−7^	63.67; 1.24 × 10^−7^

a Rows 1–6: plDDT versus lDDT and plDDT versus lDDT-Cα for round 1 monomers; rows 7–12: ModFOLD9 (MF9) score versus lDDT and ModFOLD9 score versus lDDT-Cα for round 1 monomers.

**Figure 2.**
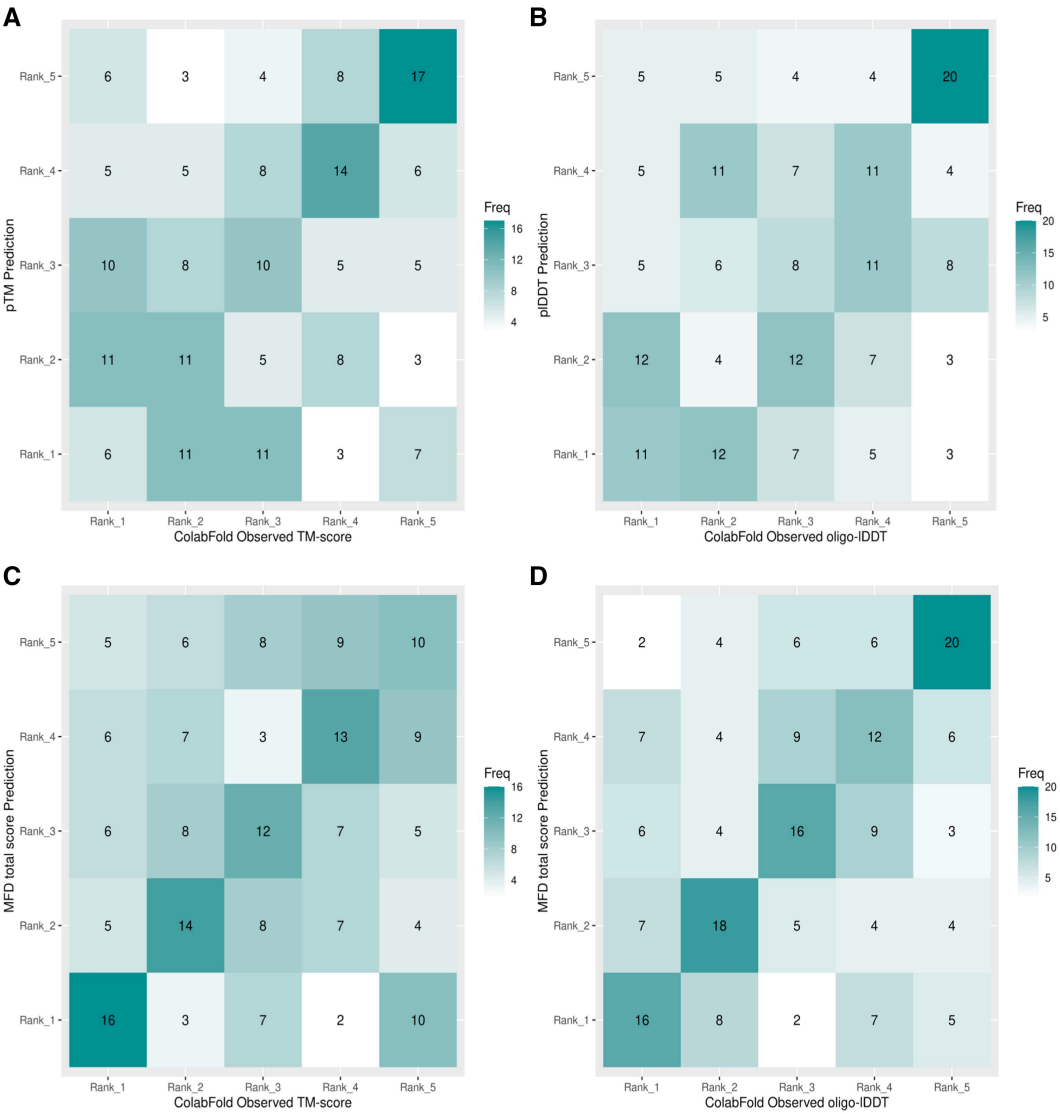
Contingency tables showing the agreement between predicted and observed score ranks. (A) pTM versus observed TM-scores and (B) plDDT versus observed oligo-lDDT scores, both for Population B1 (ColabFold multimers). (C) ModFOLDdock scores versus observed TM-scores and (D) ModFOLDdock scores versus observed oligo-lDDT scores, again, both for Population B1 (ColabFold multimers)

For the multimer population represented by Fig. 2A and B, the agreement looks appreciably less certain for both pTM and plDDT scores. For pTM (Fig. 2A), 60 models were mis-ranked by two or more places with no clear agreement between rank 1, 2, or 3 values. For plDDT (Fig. 2B), 68 models are similarly mis-ranked and there appears little agreement beyond rank 5 values. The summary statistics in Table 4 (rows 1–6), also show a reduction in mean TPR to 30.5% for pTM and 28.4% for plDDT. Both Fisher’s exact and Chi-squared *P*-values, however, remain significant suggesting a relationship between the two rank sets, although it is notable that the magnitude of the *χ*^2^ statistic has decreased for both pTM (40.26) and plDDT (51.31) suggesting a weaker association between predicted and observed ranks.

**Table 4.** Ranking agreement summary statistics for multimer models not subject to custom template recycling.^a^

Row	Test (multimers)	pTM versus TM-score	plDDT versus oligo-lDDT
1	Macro-sensitivity (TPR)	0.3052	0.2842
2	Macro-specificity	0.8263	0.8210
3	Macro-precision	0.3052	0.2842
4	Macro-accuracy	0.7221	0.7136
5	Fisher’s exact (*P*-value)	<.001	<.001
6	Chi-squared (*χ*^2^; *P*-value)	40.26; .0007	51.31; 1.41 × 10^−5^

Row	Test (multimers)	MFD versus TM- score	MFD versus oligo-lDDT
7	Macro-sensitivity (TPR)	0.3421	0.4315
8	Macro-specificity	0.8355	0.8578
9	Macro-precision	0.3421	0.4315
10	Macro-accuracy	0.7368	0.7726
11	Fisher’s exact (*P*-value)	<.001	<.001
12	Chi-squared (*χ*^2^; *P*-value)	38.42; .0013	78.94; 2.57 × 10^−10^

a Rows 1–6: pTM versus TM-Score and plDDT versus oligo-lDDT for ColabFold multimers; rows 7–12: ModFOLDdock (MFD) score versus TM-score and ModFOLDdock score versus oligo-lDDT for ColabFold multimers.

For hypothesis 2, both sets of summary statistics suggest that there is significant association between the distribution of predicted and observed ranks for both monomer and multimer model populations created via regular modelling. Accordingly, the alternative hypothesis must be accepted. ‘There is an association between the AF2 predicted and observed score ranking categories’. However, again, a qualifying statement must be added here that, despite the continuing significance of the distributions, the association appears far less robust for quaternary structure ranking by either plDDT or pTM.

### 3.3 Hypothesis 3: can AF2 model ranking accuracy be improved by independent MQA programmes?

The individual rank agreement and TPR values described above for monomer and multimer models need to be contextualized by comparison to other leading QA methods. In this section, we describe identical analysis for ranking based on predicted scores from the independent QA programmes ModFOLD9 (monomers) and ModFOLDdock (multimers).

Supplementary Figure S3, contingency tables A and B, show agreements between lDDT-Cα ranks and the predicted plDDT and ModFOLD9 ranks, respectively, for population A1 monomers. A visual comparison between the two contingency plots shows that ModFOLD9 (Supplementary Fig. S3B) has been unable to improve upon the ranking agreement between plDDT and lDDT scores in Supplementary Fig. S3A. Additionally, Table 3 (rows 7–12) shows that the ModFOLD9 TPR has reduced from 34.2%, seen for plDDT, to 26.9% (lDDT-Cα) and all other macro-averaged statistics are lower than were obtained for plDDT. The *χ*^2^ values, in agreement, have also reduced suggesting a weaker overall association between the ranks. Therefore, the closeness of the relationship has not been improved by ModFOLD9, and for hypothesis 3, in respect to monomer data, the null hypotheses must be accepted; ‘There is no difference between the independent QA and AF2 rankings as measured by the association between model rank categories’.

In contrast, a visual comparison of Fig. 2C with Fig. 2A (TM-score) and Fig. 2D with Fig. 2B (oligo-lDDT) shows that ranking agreement for multimers is stronger for ModFOLDdock scores, particularly for the oligo-lDDT score. This is further supported by the data in Table 4 (rows 7–12), showing that the TPR value has increased from 30.5% (row 1) to 34.2% (row 7) for TM-score comparisons and more appreciably from 28.4% (row 1) to 43.1% (row 7) for oligo-lDDT score. The Chi-squared values have remained similar for TM-score, however, there is an increase in the *χ*^2^ statistic from 51.31 (row 6) to 78.94 (row 12) for oligo-lDDT ranking. This increase, along with the increased TPR values, is strongly suggestive of a closer positive association between ModFOLDdock and oligo-lDDT ranking.

For hypothesis 3, with respect to multimer ranking by TM-score, there is insufficient evidence to reject the null hypothesis. ‘There is no difference between the independent QA and AF2 rankings as measured by the association between model rank categories’. However, for multimer ranking by lDDT, there is tentative evidence for accepting the alternative hypothesis. ‘Independent QA and observed score model ranks are more closely associated than AF2 and observed score model ranks’.

## 4 Discussion

In this study, plDDT has been shown to be a reliable indicator of AF2 tertiary structure model quality when using straightforward, regular modelling. Impressive Pearson correlation coefficients were obtained between plDDT and observed lDDT-Cα scores, which could not be improved upon by the independent generic QA method ModFOLD9. plDDT prediction accuracy appeared to be maintained across the scoring range and any over-prediction is likely explained by published linear relationships. Ranking of the same tertiary model population also showed an agreement between plDDT and lDDT-Cα assigned ranks, which also was not improved by ModFOLD9. Therefore, for straight forward AF2 modelling of monomers, it can be concluded that plDDT is a reliable descriptor of both model quality and ranking. That said, independent model quality estimates from methods such as ModFOLD9 are superior for comparing multiple models generated by many alternative modelling methods (McGuffin and Alharbi 2024).

Similar reliability was not maintained for multimers, however. Both pTM and plDDT showed variability for models of very similar observed quality with pTM showing a tendency for underestimation for higher quality models and both scores showed overestimation for some lower quality models. Overprediction, compared to observed scores, was statistically significant for plDDT and may have been for pTM but for the masking effect of the underprediction for higher quality models. To the best of our knowledge, this work showed for the first time the pattern of over and under-estimation of AF2-Multimer ASE scores for quaternary structure models.

For multimer model ranking accuracy, this pattern of variation resulted in lower associations with observed score ranks for both pTM and plDDT than was seen for monomer models. Of the two scores the association was weaker for plDDT-assigned ranks. ModFOLDdock did not show over-prediction to the same degree and was able to improve upon the rank agreements for plDDT, although there was insufficient evidence to draw the same conclusion for pTM. Nevertheless, there remained an unreliability in the power of pTM and plDDT to differentiate between some high and low quality multimer models created by straight-forward, regular modelling and ModFOLDdock scores represented a more reliable ranking method. As such ModFOLDdock not only remains less prone to overprediction across the model quality range, but also has the advantage that models obtained from different software, other than AF2-Multimer, can be objectively compared.

Finally, convincing evidence is presented in Supplementary Section S3.4 (S3.4.1–S3.4.4) that using the custom template option to recycle models through the AlphaFold2 algorithm resulted in much greater variability in predicted scores (both plDDT and pTM) for both tertiary structures and multimers and that the variability was more extreme for multimers. This data provides cautionary evidence that the use of AF2 and AF2-Multimer outside of their intended end-to-end operation may result in inaccurate scoring and mis-ranking of models.

## Supplementary Material

btae491_Supplementary_Data

## Data Availability

The data underlying this article are available in the article and in its online supplementary material.
