# Supplementary material for: Benchmarking of AlphaFold2 accuracy self-estimates as indicators of empirical model quality and ranking: a comparison with independent model quality assessment programmes
Source: Bioinformatics. 2024 Aug 8;40(8):btae491. doi: 10.1093/bioinformatics/btae491 (PMC11322044; doi:10.1093/bioinformatics/btae491)
Supplement: btae491_Supplementary_Data [file btae491_supplementary_data.pdf]

S1.1 Evans' description of ranking used in later versions of AlphaFold2 and ColabFold.
Ranking was calculated as a weighted combination of pTM and interface ipTM, calculated as (0.8 x ipTM + 0.2 x pTM). ColabFold v1.5.0 (Jan-2022 onwards) used the weighted ipTM-pTM score to rank multimers when using the AlphaFold2_mmseqs2, AlphaFold2_batch and colabfold_batch variants.

S2.2 Population A (CASP15 monomers).
McGuffin group's submissions for CASP15 regular tertiary structure for 26 targets: T1104, T1112, T1120, T1122, T1125, T1130, T1131, T1133, T1139, T1145, T1146, T1147, T1150, T1154, T1155, T1158, T1159, T1162, T1163, T1175, T1177, T1180, T1182, T1183, T1188 & T1194.
Population A1 represented the round 1 models (regular modelling) and these were created with a default of 12 recycles and both with and without AMBER relaxation, resulting in 20 models per target (5 unrelaxed AF2, 5 relaxed AF2, 5 unrelaxed AF2M and 5 relaxed AF2M). For a small minority of large targets memory constraints meant relaxation was not always possible resulting in fewer models. Population A2 represented the round 2 models which were subject to MultiFOLD custom template recycling (using MSA) and resulted in 10 models per target. Again, 5 of these underwent AMBER relaxation while the other 5 remained unrelaxed. In this way a maximum of 30 models were created per target.

S2.3 Population B (CASP15 multimers).
For ColabFold models (Population B1), custom template recycling and AMBER relaxation were not used and 12 recycles was used as default (Sergey Ovchinnikov, et al., 2022). The predicted scores, plDDT, pTM (and iPTM where available) were harvested directly from the server website. For MultiFOLD models (Population B2). The same pathway as outlined in section 2.2, including custom template recycling (with an MSA), was used to create these models. Only the final 5 models submitted to CASP were used for analysis and again predicted scores were collected directly from the server. For comparisons with observed scores, the official CASP15 assessor oligo-lDDT and TM-scores were downloaded from the CASP15 prediction centre results webpage. As the ModFOLDdock server participated in the CASP15 EMA experiment, predicted ModFOLDdock and ModFOLDdockR scores were also readily available for both sub populations of models. Scores for rank 1 to 5 models were collected for all multimer models for which data were available, resulting in 395 individual models across 41 targets (the ColabFold group submitted no models for three targets making a total of 38); H1106, H1111, H1114, H1129, H1134, H1135, H1137 (MultiFOLD only), H1140, H1141, H1142, H1143, H1144, H1151, H1157, H1166, H1167, H1168, H1171, H1172, H1185, T1109, T1110, T1113, T1115 (MultiFOLD only), T1121, T1123, T1124, T1127, T1132, T1153, T1160, T1161, T1170, T1173, T1174, T1176, T1178, T1179, T1181, T1187 and T1192 (MultiFOLD only).

S2.4 Population C (recycled monomers).
The AlphaFold2 Rank 1 models were downloaded from the CASP14 website for the following 20 CASP14 FM targets: T1027, T1029, T1031, T1033, T1037, T1039, T1040, T1041, T1042, T1043, T1047s1, T1047s2, T1055, T1058, T1064, T1074, T1090, T1093, T1094, T1096. Again, as described in section S2.3, observed quality assessment scores were generated using the downloadable versions of TM-score and lDDT score. To affect the recycling, model PDB files were converted to mmCIF format using the RSCB PDB MAXIT suite of programs (https://mmcif.pdbj.org/converter). These were then submitted to the Google Colaboratory hosted ColabFold (release 3, v1.3.0 [4-Mar-2022]) as custom templates along with their respective amino acid sequences. ColabFold was run twice per model (both MSA and single-sequence modes), and, within each mode, the model was submitted four times for 1, 3, 6 and 12 recycles. ColabFold settings used were: Template_mode: custom; msa_mode: MMseqs2 (UniRef+Environmental) OR single sequence; pair_mode: unpaired+paired; model-type: auto; num_recycles: 1, 3, 6, 12 (selecting "auto" from the model type defaulted to the original pre-CASP14 AF2 model). Amber relaxation was not enabled. Models created for each ColabFold run were collected along with their predicted pTM and plDDT scores and then rescored with TM-score and lDDT as described above.

The same logic was employed for nonAF2 CASP14 models. These were selected from the same 20 FM targets for the next five best-ranked groups beneath AlphaFold2 at CASP14. These were Baker (473), Baker-experimental (403), Feig-R2 (480), Zhang (129) and tFold_human (009). To ensure consistency in terms of globular fold similarity, only models with a TM-score >=0.45 were selected and this resulted in a total of 47 individual models.
The full list of models used is:
T1029TS009_1-D1, T1031TS009_1-D1, T1033TS009_1-D1, T1037TS009_1-D1, T1041TS009_1-D1, T1042TS009_1-D1, T1043TS009_1-D1, T1049TS009_1-D1, T1090TS009_1-D1, T1031TS129_1-D1, T1037TS129_1-D1, T1040TS129_1-D1, T1041TS129_1-D1, T1042TS129_1-D1, T1049TS129_1-D1, T1074TS129_1-D1, T1090TS129_1-D1, T1096TS129_1, T1027TS403_1-D1, T1031TS403_1-D1, T1033TS403_1-D1, T1037TS403_1-D1, T1039TS403_1-D1, T1041TS403_1-D1, T1042TS403_1-D1, T1043TS403_1-D1, T1049TS403_1-D1, T1090TS403_1-D1, T1096TS403_1, T1031TS473_1-D1, T1033TS473_1-D1, T1037TS473_1-D1, T1039TS473_1-D1, T1041TS473_1-D1, T1042TS473_1-D1, T1043TS473_1-D1, T1049TS473_1-D1, T1074TS473_1-D1, T1090TS473_1-D1, T1031TS480_1-D1, T1037TS480_1-D1, T1041TS480_1-D1, T1042TS480_1-D1, T1049TS480_1-D1, T1074TS480_1-D1, T1090TS480_1-D1, T1096TS480_1.
Models were downloaded from the CASP14 website, scored with TM-score and lDDT and modelled with the MSA option in the same way as described for AF2 models. Single sequence modelling was carried out using release v1.3.0 of Localcolabfold (Mirdita *et al.*, 2022) installed on our own server, to overcome the Google Colaboratory GPU restrictions in the time available. The equivalent Localcolabfold settings were used: msa-mode: single_sequence; model-type: auto; rank: plddt; pair-mode: unpaired+paired; templates: --custom-template-path. The resulting rank 1-5 models were collected along with their plDDT and pTM scores and rescored against the native structure to produce a set of observed lDDT and TM-scores.

S2.5 Population D (recycled multimers)

Some of multimer targets were too large to recycle through AF2-Multimer (training was limited to models up to 1536 residues and the algorithm can experience memory issues with models of more than a few thousand residues (Bryant *et al.*, 2022) and therefore the targets used in this set were limited by size to: H1045, H1065, H1072, T1032, T1054, T1070, T1073, T1078, T1083, T1084. Again, top-ranked models were used as the custom templates and were subjected to recycling (1, 3, 6 and 12) using ColabFold (MSA and SS modes) in the same way as described for the monomer structures above. The resulting 50 rank 1-5 models were then collected along with their plDDT and pTM scores. Observed scores were obtained by assessing each model against their relevant native structures using the OpenStructure and MM-Align (Mukherjee and Zhang, 2009) programs to obtain observed oligo-lDDT and TM-scores respectively.

S2.6 Handling of contingency table data, multimer pTM scores and the model ranking procedure.

Multimer models created by AlphaFold2 variants are, by default, ranked by pTM rather than plDDT. As stated in the introduction there is a slight difference in the calculation of the pTM-based ranking between versions of ColabFold. In AF2-Multimer and in later versions of ColabFold (v1.5.0) ranking is calculated based on a ratio of 0.8 * ipTM + 0.2 * pTM (Richard Evans, 2021), whereas in earlier versions, ranking is calculated on pTM score alone. As some multimer models in this population were created with ColabFold v1.3 and some with v1.5 there was potentially heterogeneous ranking across the model population, and it was necessary to allow for this when comparing ranks. To this end, multimer models were routinely re-ranked by pTM score before comparison with observed rankings. The procedure for deriving model ranks in R consisted of ranking each individual set of 5 related models, i.e., models output from a single run of AlphaFold2 modelling, using the statement *rank <score>, ties.method ="random"* where <score> can be replaced with any of the predicted or observed scores as necessary. This was applied to observed score ranking but also to ranking by pTM for the reasons explained above. In this way any differences in the way the AlphaFold2 algorithm originally ranked the data were negated and the ranks were assigned uniformly across all populations.

Multi-factor contingency tables to display ranking comparisons were created in R using the caret package with the confusionMatrix() command and four further statistical measures were used to assess relatedness. Sensitivity, specificity, precision, and accuracy were calculated for individual rank classes (1, 2, 3, 4 and 5) and, to construct meaningful comparisons between the contingency tables, macro-averaged versions of these statistics were calculated as mean values across all categories for each table.

Fisher's exact test is often used for smaller sample sizes (single contingency table cells of less than 5) or where independence of observations cannot be guaranteed and, while the concept of independence holds for the assignment of ranks based on predicted and observed scores, some tables do have low figures in individual cells. As regards multi-contingency tables (larger than 2x2), no clear distinction between the two tests could be found other than Chi-squared may run into problems with very sparse data and Fisher's can become computationally intensive for large tables.

As ranking data is categorical, it is possible to assess the association between the predicted model ranks and observed model ranks using the Chi-squared and Fisher's exact tests, where P-values <0.05 would suggest relatedness between distributions. It was decided that both tests would be run as a check for each other, i.e., agreement between the two tests would confer confidence in the result. A Monte Carlo resampling method (simulate.p.value) with default simulations of 2000 was used for the Fisher's exact test to allow a more robust estimate of the p-value and prevent any computational overheads which can occur when this test is applied to larger contingency tables (Crawley, 2015). Analysis was performed using R version 3.6.3 running in R-studio.

S2.7 Oligo-lDDT, ModFOLDdock and ModFOLD9 score calculations

The local Distance Difference Test (lDDT) is a superposition-free, local, all atom score with a range of 0-1. It expresses the fraction of contacts shared or conserved between a model and its native structure regardless of orientation. Oligo-lDDT is the multimer equivalent which uses the QS-score chain mapping routine to identify intra and inter-chain contacts prior to calculating the test score. The score penalises both deficiency of atoms and incorrect stoichiometry in the model structure and, while this is a good measure of, for example, domain or individual chain similarity, it can be argued that it gives little impression of the orientation of one domain to the next or one chain to another, for the oligo version.

The ModFOLDdock server combines seven individual scoring methods: ModFOLDIA, our own clustering interface accuracy score; DockQJury, a clustering approach based on the DockQ score; QSscoreJury and QSscoreOfficialJury, clustering approaches using QS-scores; lDDTOfficialJury, clustering using lDDT scores; voronota-js-voromqa, the Voronoi tessellation score and the CDA-score, our contact distance agreement score. More details of these scores can be found in our latest IntFOLD7, MultiFOLD and ModFOLDdock server paper which is accessible via the MultiFOLD server available at: https://www.reading.ac.uk/bioinf/ModFOLDdock/.

The ModFOLD9 server is the latest version of our tertiary structure hybrid clustering and single-model MQA program ModFOLD. It combines numerous clustering scores with pure and quasi-single model scores to form an overall consensus score. The exact details are explained in our latest ModFOLD9 server paper which can be access via the ModFOLD server at: https://www.reading.ac.uk/bioinf/ModFOLD/.

S3. Supplementary results figures and tables

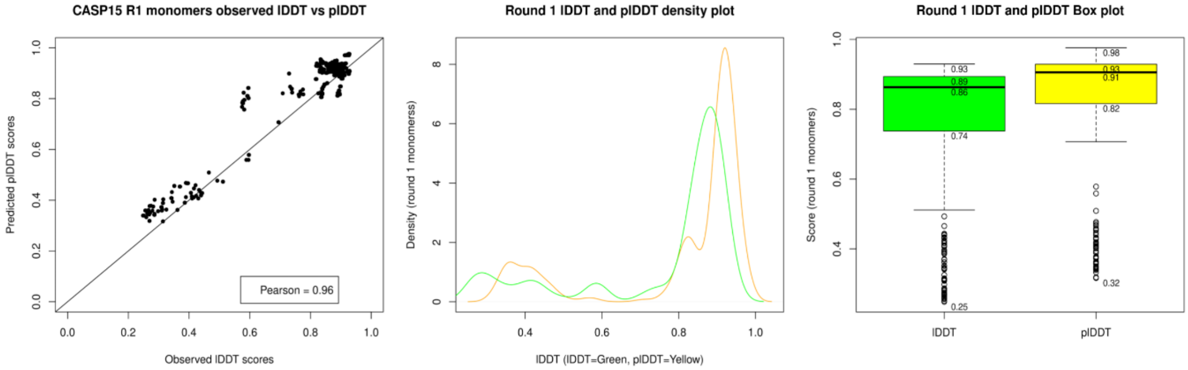

Figure S1. Plots of plDDT versus observed all atom lDDT for round 1 monomers population A1. Scatter plot (left), density plot (middle) and boxplot (right). plDDT is rescaled to the 0-1 range.

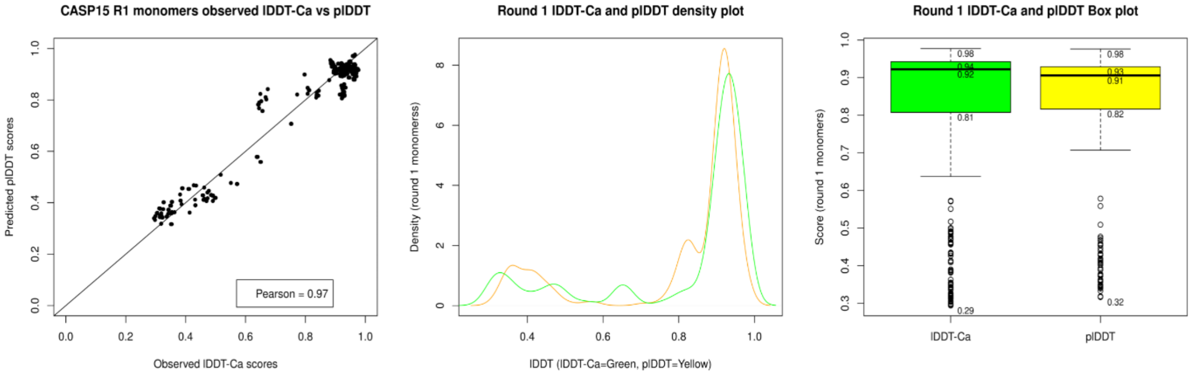

Figure S2. Plots of plDDT versus observed lDDT-Cα for round 1 monomers population A1. Scatter plot (left), density plot (middle) and boxplot (right). plDDT is rescaled to the 0-1 range.

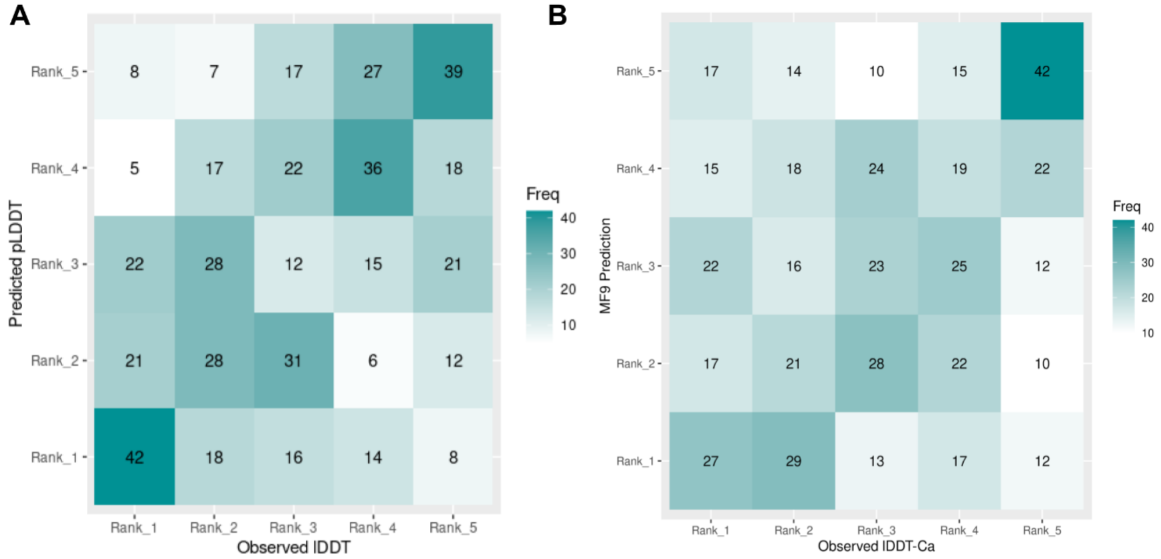

Figure S3. Contingency tables showing the agreement between predicted and observed score ranks. A. plDDT versus observed lDDT-Cα scores for Population A1 (round 1 monomers). **B.** ModFOLD9 scores versus observed lDDT-Cα also for Population A1 (round 1 monomers).

S3.4 Results for custom template recycled models. Hypothesis 4. Is the accuracy of predicted scores affected by custom template recycling?

To answer this question data is presented from the four model populations which underwent custom template recycling. For monomers this is Population A2 (CASP15 round 2 monomers) and Population C (recycled monomers), for multimers it is Population B2 (CASP15 MultiFOLD group multimers) and Population D (recycled multimers). It would be logical to start with the data for populations A2 and B2 because these two groups can be directly compared to their unrecycled counterparts, i.e. Population A2, the CASP15 round 2 monomers (recycled) can be directly compared with the Population A1 CASP15 round 1 monomers (unrecycled) which were discussed in section 3.1.1 and Population B2, the MultiFOLD group multimers (recycled) can be directly compared to the Population B1 ColabFold group multimers (unrecycled) which were discussed in section 3.1.2. Populations C and D have no direct comparisons and so will be discussed last to provide support of the population A and B data.

S3.4.1 Population A2 (CASP15 round 2 monomers).

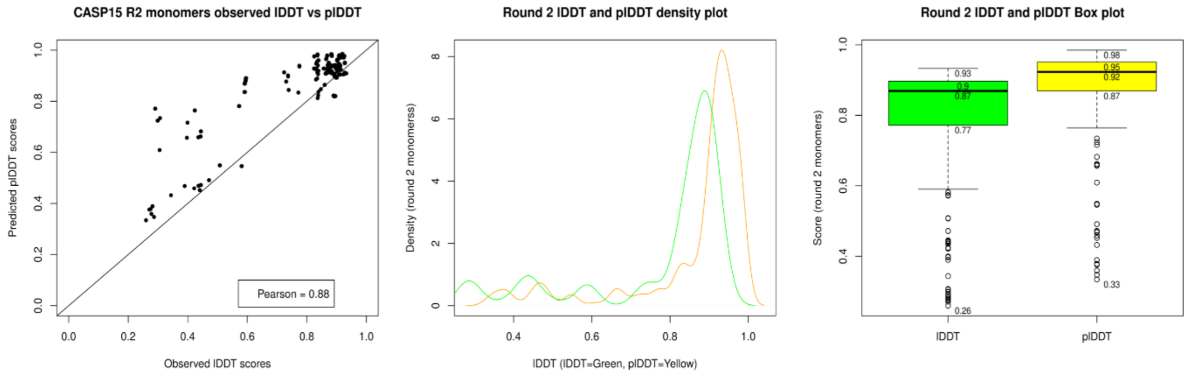

Figure S4. Plots for plDDT versus observed lDDT for Population A2 (CASP15 round 2 monomers). A scatter plot (left), density plot (middle) and boxplot (right). For all plots plDDT has been rescaled to fit the 0-1 lDDT range.

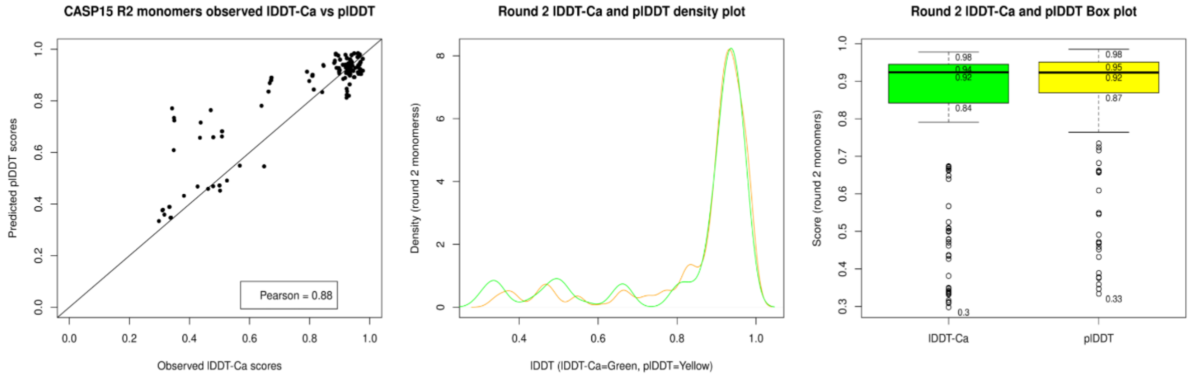

Figure S5. Plots for plDDT versus observed lDDT-Cα for Population A2 (CASP15 round 2 monomers). A scatter plot (left), density plot (middle) and boxplot (right). For all plots plDDT has been rescaled to fit the 0-1 lDDT range.

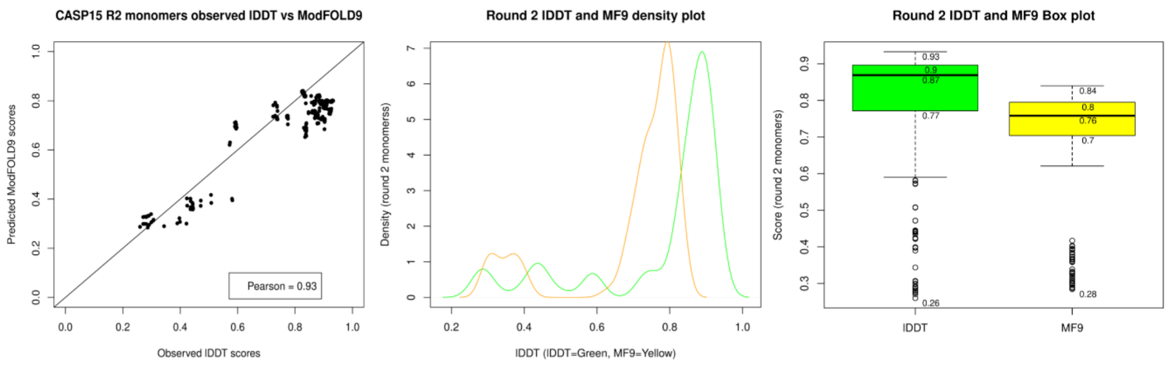

Figure S6. Equivalent plots of ModFOLD9 score versus observed lDDT for Population A2 (CASP15 round 2 monomers). A scatter plot (left), density plot (middle) and boxplot (right). For all plots plDDT has been rescaled to fit the 0-1 lDDT range.

Comparing the data from Figure S4 directly with that for the round 1 monomers in Figure 2, it is clear that there is a wider spread of data in the scatter plot in Figure S4 with an increase in plDDT scores, which are reflected in the density plot and the boxplot. Figure S5, for lDDT-Cα scores, shows a similar spread in the scatter plot but accompanied by a less noticeable difference between the plDDT and lDDT-Cα distributions in the density and boxplot. Wilcoxon signed rank tests for significance in Table S1 (below), however, reveal that the difference between the plDDT and lDDT-Cα score is significant as is the difference between the round 2 monomer plDDT scores and their round 1 counterparts.

Table S1. Wilcoxon statistics for Population A2 round 2 monomers. (significant figures in bold).

Row	Scores compared	Independence and distribution symmetry	p-value
1	R2 plDDT and lDDT-Cα	Paired; 2-sided test	**0.0001**
2	R2 plDDT and lDDT-Cα	Paired; 1-sided test, plDDT > lDDT-Cα	**$5.83x10^{-5}$**
3	R2 plDDT and R1 plDDT	Unpaired; 2-sided	**$1.293x10^{-9}$**
4	R2 plDDT and R1 plDDT	Unpaired; 1-sided, R2 > R1	**$6.465x10^{-10}$**
5	R2 lDDT-Cα and R1 lDDT-Cα	Unpaired; 2-sided	0.1255

Table S1, row 1, shows that according to a paired 2-sided Wilcoxon test there is a significant difference between plDDT and lDDT-Cα observed scores for round 2 monomers and, further to this, the results of a paired 1-sided test in row 2 show that that plDDT scores are significantly higher. These findings agree with the scatter plots in Figures S4 and S5 showing over-prediction in mid-quality models which was not present in the round 1 data. Notably, the over-prediction is also absent from the equivalent round 2 ModFOLD9 scatter plot shown in Figure S6. This is good evidence that overprediction of plDDT occurs in monomer models with custom template recycling.

To further test this, a 2-sided Wilcoxon test was used to directly compare round 1 and round 2 monomer plDDT scores (row 3) and this showed a significant difference between the two scores, evidenced by a p-value of $1.293x10^{-9}$. Further, it was established that the round 2 monomer scores were significantly higher than those for round 1, evidenced by a p-value of $6.465x10^{-10}$ from the 1-sided test in row 4. Importantly, there was no such difference between the equivalent round 1 and 2 monomer observed lDDT-Cα scores as shown by the p-value of 0.1255 (row 5 of the table) meaning that round 1 and 2 monomer models were not significantly different in quality.

It is therefore reasonable to conclude that these prediction errors have been introduced by custom template recycling and, for hypothesis 4 in respect to monomer models, the alternative hypothesis can be accepted, i.e., *AF2 predicted scores following custom template modelling show greater variation than scores from regular modelling, when compared to equivalent observed scores.*

S3.4.2 Population B2 (CASP15 MultiFOLD multimers).

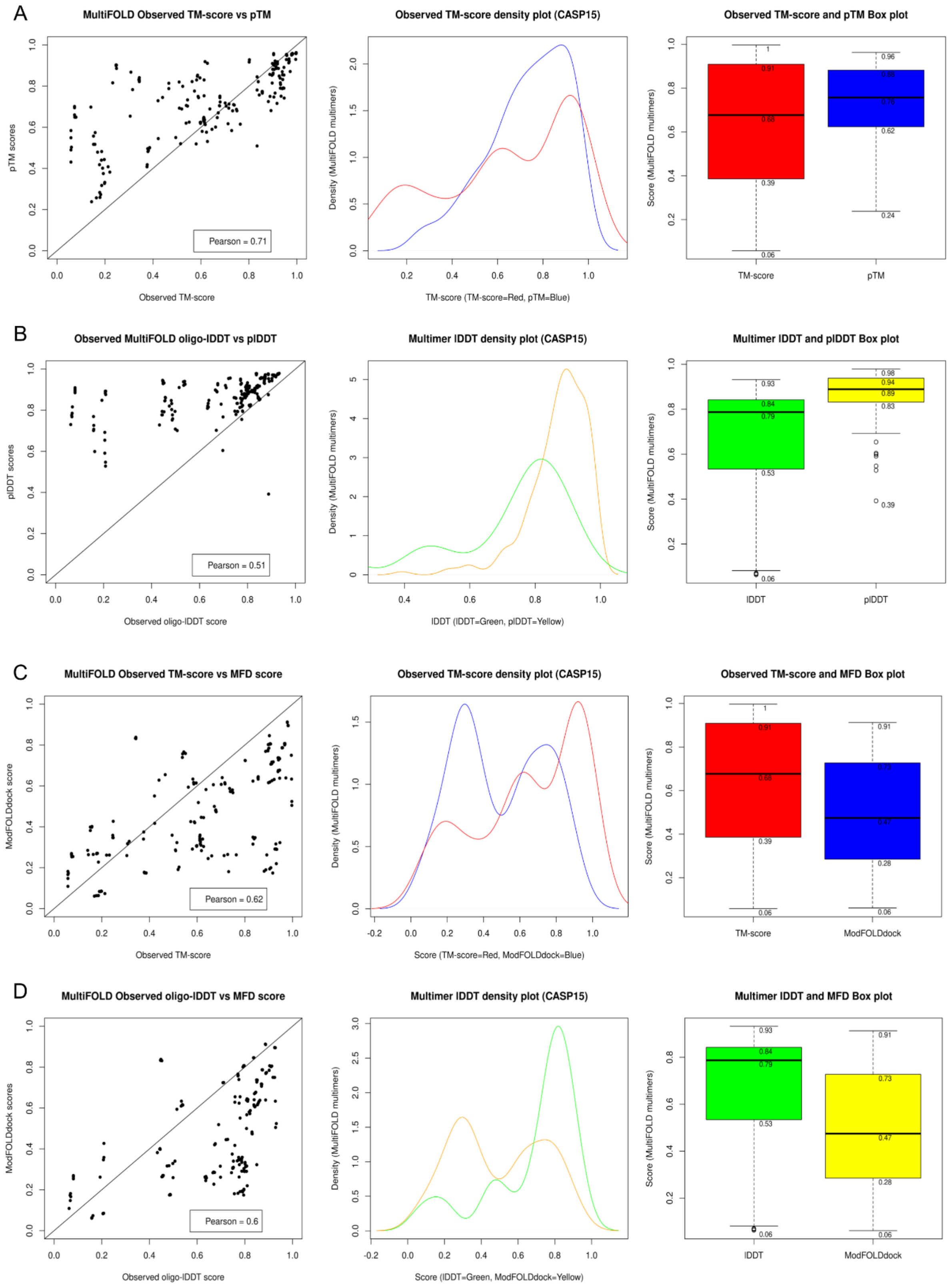

Figure S7. Plots for Population B2 (MultiFOLD multimers). scatter plots (left), density plots (middle) and box plots (right) for. Panel A: pTM versus observed TM-score. Panel B: plDDT versus observed CASP oligo-lDDT. Panel C: Comparison plots for ModFOLDdock score versus TM-score, Panel D Comparison plots for ModFOLDdock versus oligo-lDDT.

The plots in Figure S7, panels A and B, can be directly compared to Figures 1A and 1B for ColabFold multimers in section 3.1.2. Considering the plots in panel A for TM-scores, the spread of points in the scatter plot is again noticeably greater than that shown in Figure 4. Further, although the mean observed TM-score in the boxplots reduces from 0.745 (Figure 4) to 0.68 across the two populations, the equivalent mean pTM rises from 0.72 to 0.76. Secondly, considering panel B for lDDT scores in a similar way, the scatter plot again shows an increase in the spread of data compared to its equivalent in Figure 5. and there is also a marked shift to the right in plDDT when comparing the density plots, and a corresponding increase in mean plDDT score shown in the boxplot. These changes suggest a similar overprediction to that seen for monomers is also occurring for multimers which have been subject to custom template recycling. For comparison, the scatter plots in panel C showing ModFOLDdock scores versus both observed TM-score and oligo-lDDT scores for the same population, show little evidence of sustained overprediction. If anything, ModFOLDdock appears to suffer from a tendency for under-prediction of these models.

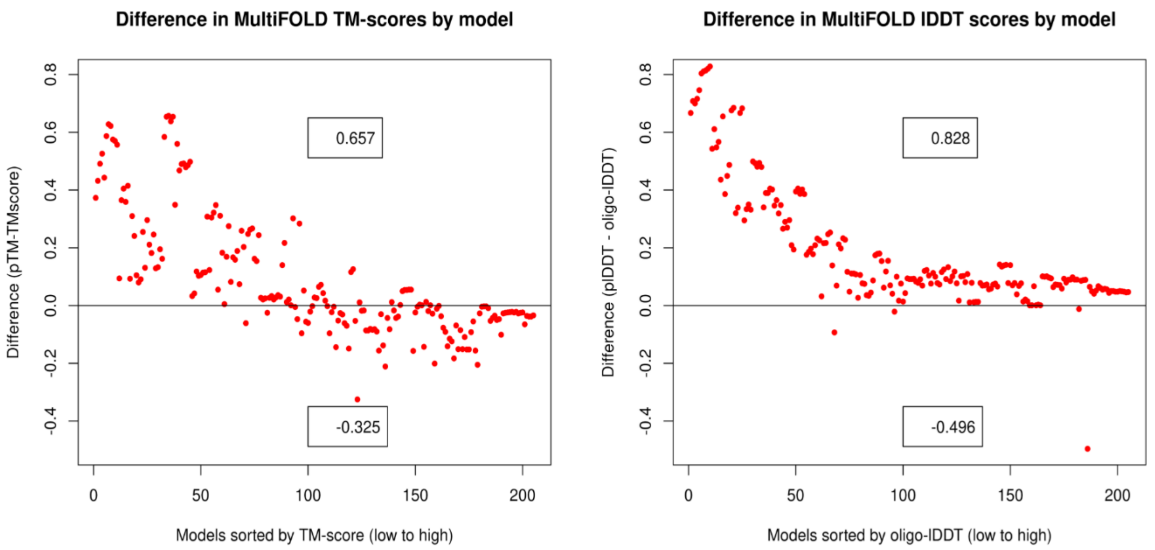

Figure S8. Plots to show variation between predicted and observed scores for Population B2 (MultiFOLD multimers). Left, pTM versus TM-score and right, plDDT versus oligo-lDDT. Plots are equivalent to those in Figure 6 for ColabFold multimers.

The relationships suggested in Figure S7, panels A and B, are more clearly shown by the two variation plots in Figure S8. In agreement with Figure 6, both plots show overprediction of scores for lower quality models with maximum and minimum differences of +0.657 and -0.325 respectively for pTM score and a maximum difference of +0.828 for plDDT score. Although the maximum and minimum deviation in the data for pTM score are almost identical to those from Figure 6, the maximum deviation in plDDT scores has increased from 0.747 to 0.828. Also, upon visual comparison of the two pairs of plots it is clear that the number models in the over-predicted regions in Figure S8 has increased over those in Figure 6 despite a similar number of models (205 and 190 respectively). Wilcoxon signed rank tests were again used to quantify these differences in terms of significance and the results are presented in Table S2 below.

Table S2. Wilcoxon tests for Population B2 MultiFOLD multimers and Population B1 ColabFold multimers. (significant figures in bold).

Row	Scores compared	Independence and distribution symmetry	p-value
1	MultiFOLD plDDT and oligo-lDDT	Paired; 1-sided test, plDDT > oligo-lDDT	**$2.20x10^{-16}$**
2	MultiFOLD pTM and TM-score	Paired; 1-sided test, pTM > TM-score	**$1.46x10^{-5}$**
3	MultiFOLD and ColabFold plDDT	Unpaired; 1-sided, MultiFOLD > ColabFold	**$7.193x10^{-8}$**

4	MultiFOLD and ColabFold oligo-lDDT	Unpaired; 2-sided.	0.283
5	MultiFOLD and ColabFold pTM	Unpaired; 1-sided; MultiFOLD > ColabFold	**0.014**
6	MultiFOLD and ColabFold TM-score	Unpaired; 2-sided.	0.252

Table S2, rows 1 and 2 confirm that both predicted plDDT and pTM scores are significantly greater than their observed counterparts (lDDT and TM-scores) for MultiFOLD multimers as evidenced by p-values of 2.20×10^{-16} for plDDT versus oligo-lDDT and 1.46×10^{-5} for pTM versus TM-score. Furthermore, there is confirmation that MultiFOLD plDDT (row 3) and pTM (row 5) scores are significantly greater than the equivalent predicted scores for ColabFold multimers but, importantly, there is no significant difference between the equivalent two sets of observed scores (row 4 for oligo-lDDT and row 6 for TM-score). This again shows that, for a similar set of models based on the same CASP targets, both sets of observed scores are similar but both predicted pTM and plDDT scores are significantly different and are higher in both cases for the group subject to custom template recycling.

Therefore, with respect to multimers, the alternative hypothesis must again be accepted, i.e., *AF2 predicted scores following custom template modelling show greater variation than scores from regular modelling, when compared to equivalent observed scores.*

S3.4.3 Population C (recycled monomers).

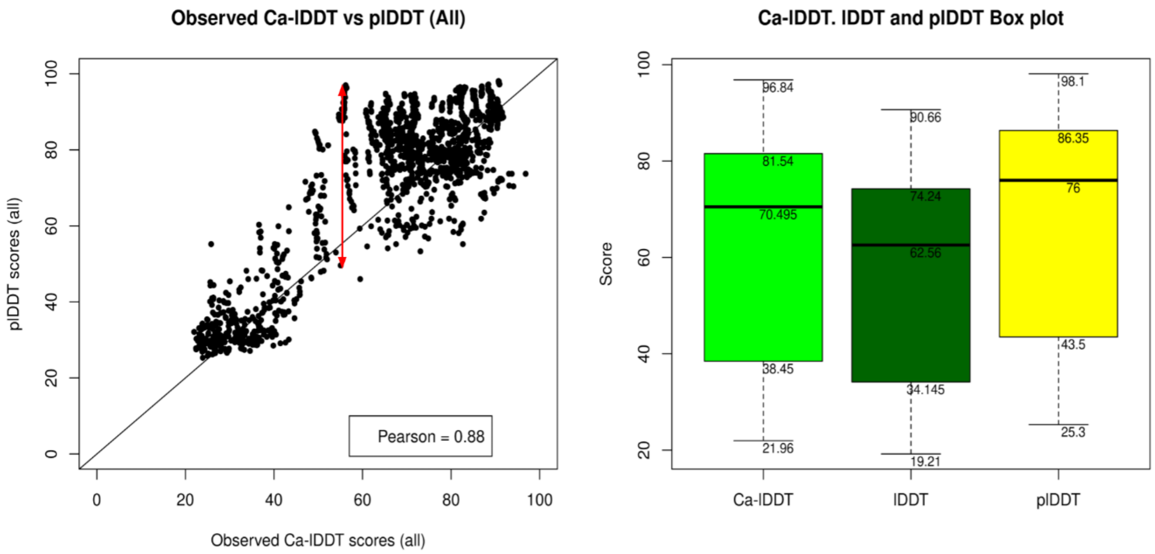

Figure S9. Plots for plDDT versus observed lDDT-Cα for population C (recycled monomers). Left, a scatter plot showing the spread of data and right, a boxplot comparing the distribution of lDDT-Cα, lDDT and plDDT scores for the same population. lDDT and lDDT-Cα have been rescaled to the 0-100 range.

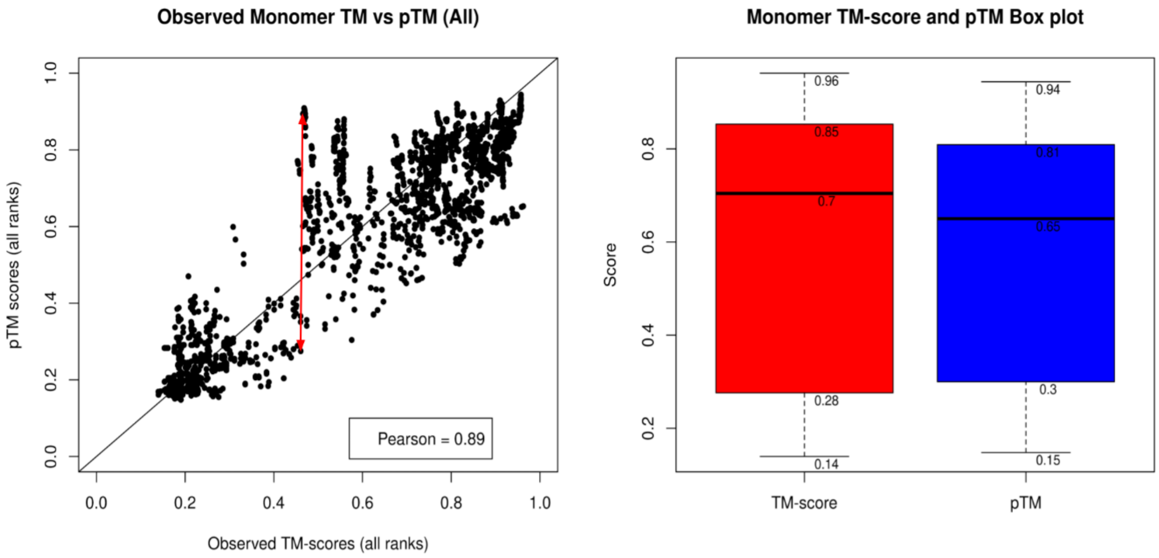

Figure S10. Plots for pTM versus observed TM-score for population C (recycled monomers). Left, a scatter plot showing the spread of data and right, a boxplot for both scores from the same population.

The scatter plots in Figures S9 and S10 show Pearson correlation coefficients of 0.87 and 0.89 respectively between predicted and observed scores. Although these correlations appear very respectable, both plots show a pronounced spread in the data with a high proportion of outliers. The red bars on each scatter plot show the potential degree of variation in predicted scores for models with similar observed scores. For an observed score of approximately 0.5, predicted plDDT scores range from approximately 0.5 to 0.9 (Figure S9) and pTM scores range from approximately 0.3 to 0.9 (Figure S10).

These results strongly support the hypothesis that using custom template recycling appears to produce a much higher degree of variability both plDDT and pTM scores.

When the models in this population were split into those recycled using an MSA and those recycled using only single sequence (SS) mode, there was found to be a noticeable difference in the quality profile between the two groups. Figures S9A and S9B below show the effect of recycling separately using MSA mode (Figure S9A) and single sequence mode (S9B) as determined by comparing plDDT and lDDT-Cα scores.

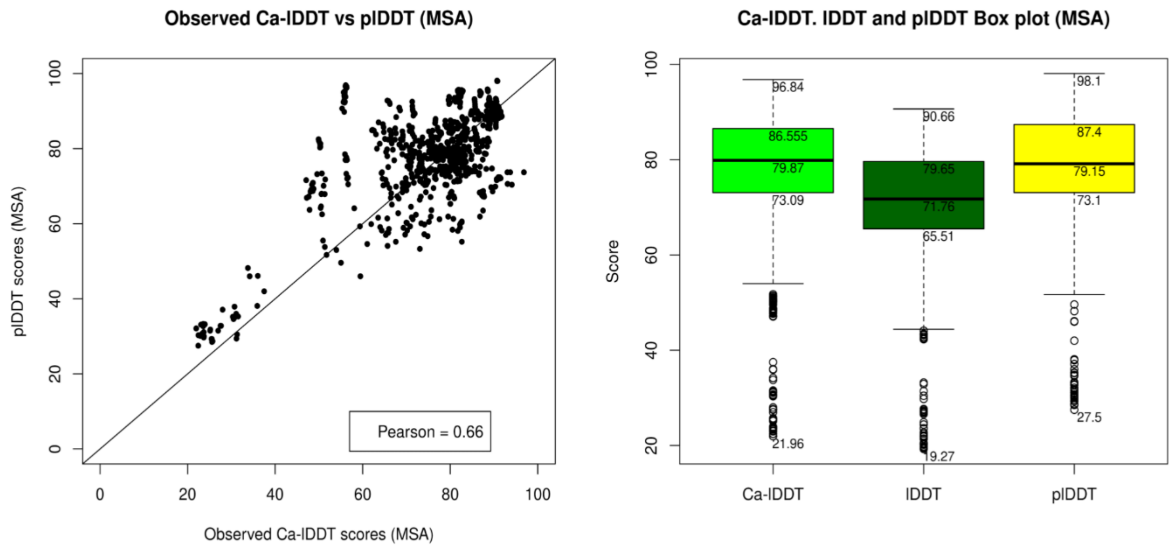

Figure S9A. Plots for plDDT versus observed lDDT-Cα for population C (recycled monomers) recycled using MSA mode. Left, a scatter plot showing the spread of data and right, a boxplot comparing the distribution of lDDT-Cα, lDDT and plDDT scores for the same population. lDDT and lDDT-Cα have been rescaled to the 0-100 range.

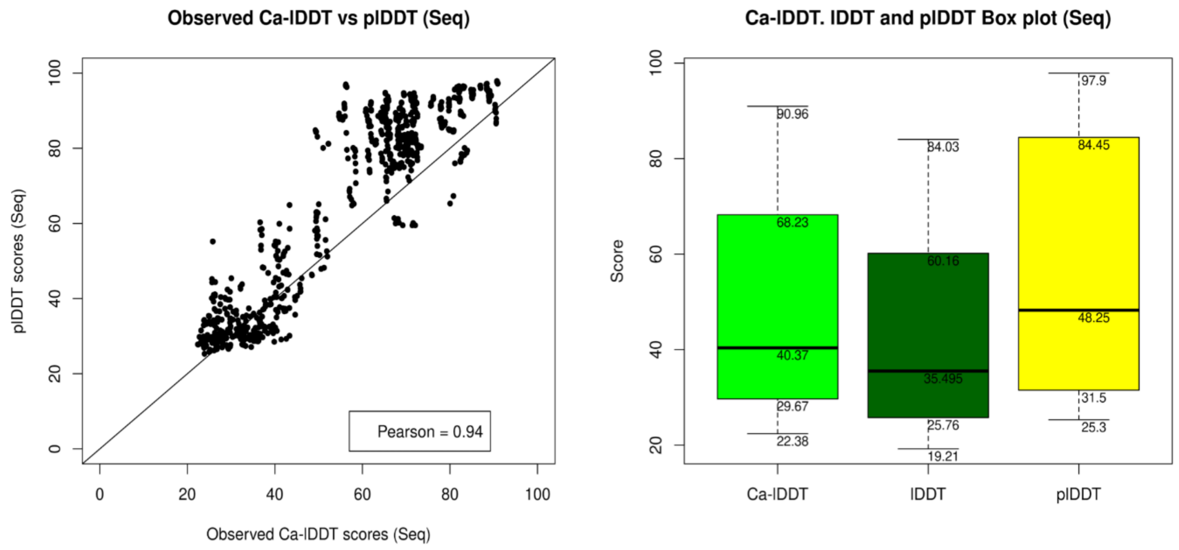

Figure S9B. Plots for plDDT versus observed lDDT-Cα for population C (recycled monomers) recycled using single sequence (labelled Seq) mode. Left, a scatter plot showing the spread of data and right, a boxplot comparing the distribution of lDDT-Cα, lDDT and plDDT scores for the same population. lDDT and lDDT-Cα have been rescaled to the 0-100 range.

Although the scatter plots look similar, there appears to be a difference in the box plots across Figures S9A and S9B when lDDT-Cα is compared with plDDT and it appears that there is a larger spread for plDDT scores using single sequence mode. This was investigated further by calculating the squared error and the mean squared error (MSE) between plDDT and lDDT-Cα for both recycle modes. MSE for the MSA group was calculated as 202.97 whereas MSE for the single sequence group was higher at 303.74. The squared error values were then compared using a Wilcoxon rank sum test and the results are shown below in Table S3. These results show that there was a greater plDDT over-prediction for single sequence models than for MSA models). This difference was significant when the

calculated squared errors were compared by a Wilcoxon rank sum test shown in table S3, row 1. However, it remained true that plDDT scores were significantly higher than lDDT-Cα scores across both MSA and single sequence populations as measured by a paired Wilcoxon signed rank test shown in table S3, rows 2 and 3.

Table S3. Population C. Wilcoxon rank sum test results for the comparison of the squared error values calculated as (plDDT – lDDT-Cα)^2 between MSA and SS mode (row 1). Values for a Wilcoxon signed rank test between plDDT and lDDT-Cα scores within each subpopulation; MSA mode (row 2) and SS mode (row 3) (significant figures in bold).

Row	Scores compared	Independence and distribution symmetry	p-value
1	Squared Errors for MSA and SS groups.	Unpaired; 2-sided	**2.20x10^{-16}**
2	plDDT and lDDT-Cα scores (MSA)	Paired; 1-sided test, plDDT > lDDT-Cα	**2.20x10^{-16}**
3	plDDT and lDDT-Cα scores (SS)	Paired; 1-sided test, plDDT > lDDT-Cα	**2.20x10^{-16}**

Similar analysis was performed using the pTM and TM-scores and equivalent plots are shown below in Figures S10A and S10B .

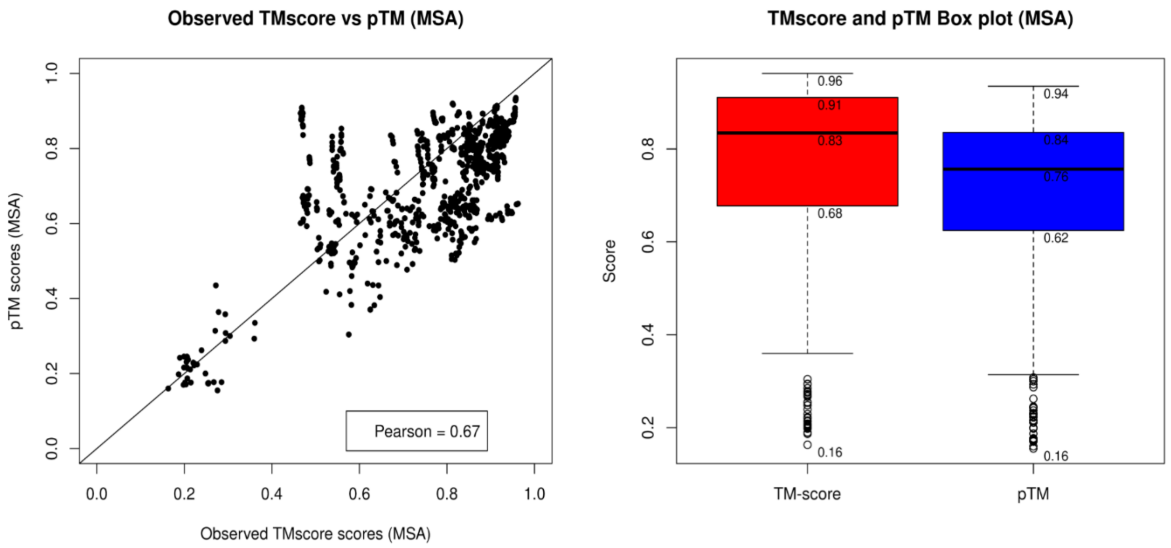

Figure S10A. Plots for pTM versus observed TM-score for population C (recycled monomers) recycled using MSA recycling. Left, a scatter plot showing the spread of data and right, a boxplot for both scores from the same population.

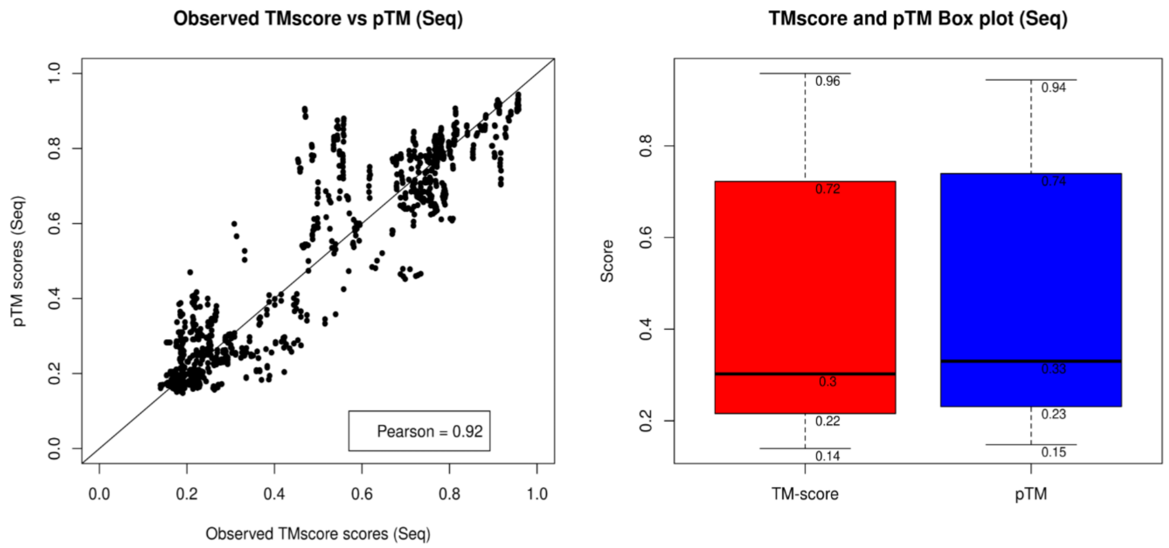

Figure S10B. Plots for pTM versus observed TM-score for population C (recycled monomers) recycled using single sequence (labelled Seq) recycling. Left, a scatter plot showing the spread of data and right, a boxplot for both scores from the same population.

Again, the two recycle modes were compared by calculating the squared error between pTM and TM-scores and the results of a Wilcoxon rank sum test are shown in Table S4 along with the results of a Wilcoxon signed rank test comparing pTM with TM-score within each group. For the MSA group, the MSE was calculated as 0.0224 whereas the equivalent MSE was 0.0106 for the single sequence group. This shows that the opposite was seen for TM-score than was observed for lDDT score; that the MSA models had a higher MSE between predicted and observed scores than the single sequence models.

Table S4. Population C. Wilcoxon rank sum test results for the comparison of the squared error values calculated as (pTM – TM-score)^2 between MSA and SS mode (row 1). Values for a Wilcoxon signed rank test between pTM and TM-scores within each subpopulation; MSA mode (row 2) and SS mode (row 3) (significant figures in bold).

**Row**	**Scores compared**	**Independence and distribution symmetry**	**p-value**
1	Squared Errors for MSA and SS groups.	Unpaired; 2-sided	**$2.20\text{x}10^{-16}$**
2	pTM and TM-score scores (MSA)	Paired; 1-sided test, pTM < TM-score	**$2.20\text{x}10^{-16}$**
3	plDDT and lDDT-Cα scores (SS)	Paired; 1-sided test, pTM > TM-score	**0.0005**

Another difference shown in Table S4 is that for MSA recycled models TM-score overall was greater than pTM score, whereas for single sequence models pTM was overall greater than TM-score.

S3.4.4 Population D (recycled multimers).

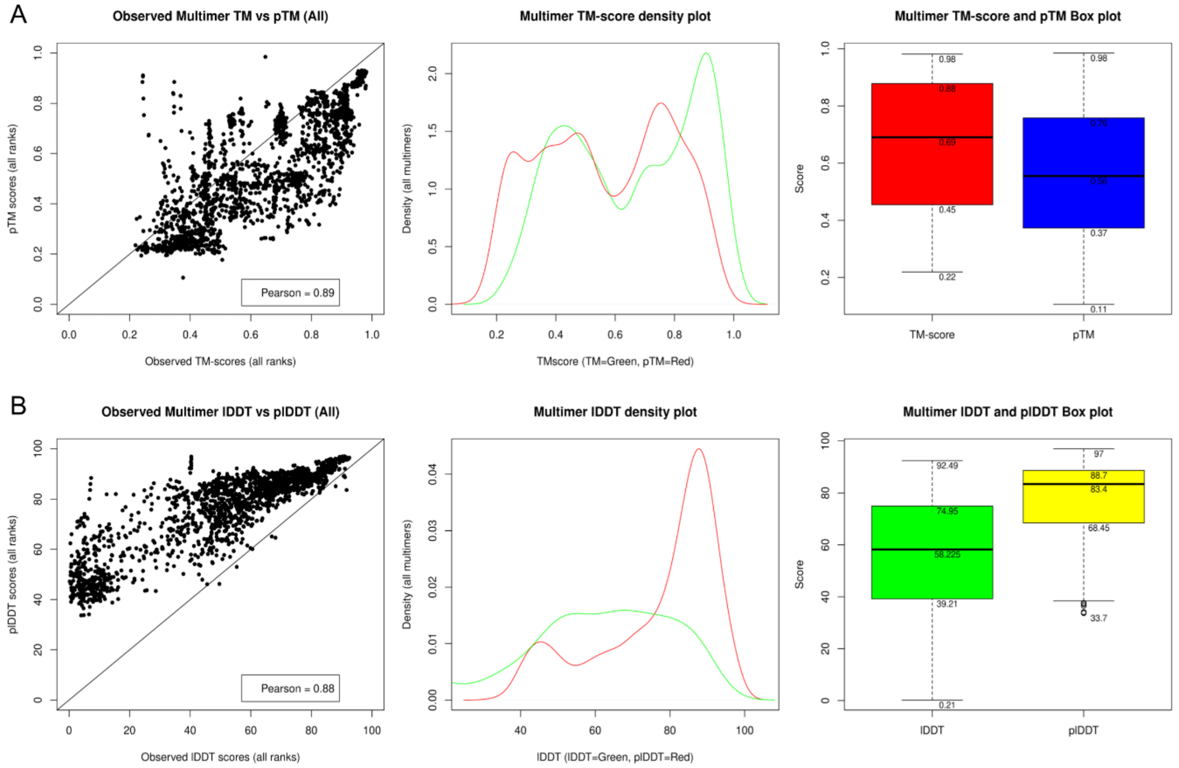

Figure S11. Plots for Population D (Recycled multimers). Scatter plots (left), density plots (middle) and box plots (right). Panel A: pTM versus observed TM-score and panel B: plDDT versus observed CASP oligo-lDDT.

Figure S11 shows a similar spread of data to that seen in Figures S9 and S10. Panel A again shows a tendency for multimer pTM over and under-prediction meaning a high variation in predicted pTM score for models with similar observed scores. In panel B, all three plots demonstrate a high tendency for plDDT over-prediction and again, this is more pronounced for mid to lower quality models.

As both population C and D were subject to up to 12 recycles and were entirely created via custom template recycling, these results support the hypotheses drawn above for population A and B, that using custom template recycling produces a higher degree of variability in AlphaFold2 predicted scores for both monomer and multimer models and that this effect is more pronounced for multimers.

Again, for population D the difference between MSA and single sequence recycling was examined and Figures S12 and S13 show plots equivalent to those in Figure S11 for pTM versus TM-scores (S12) and plDDT versus oligo-lDDT (S13).

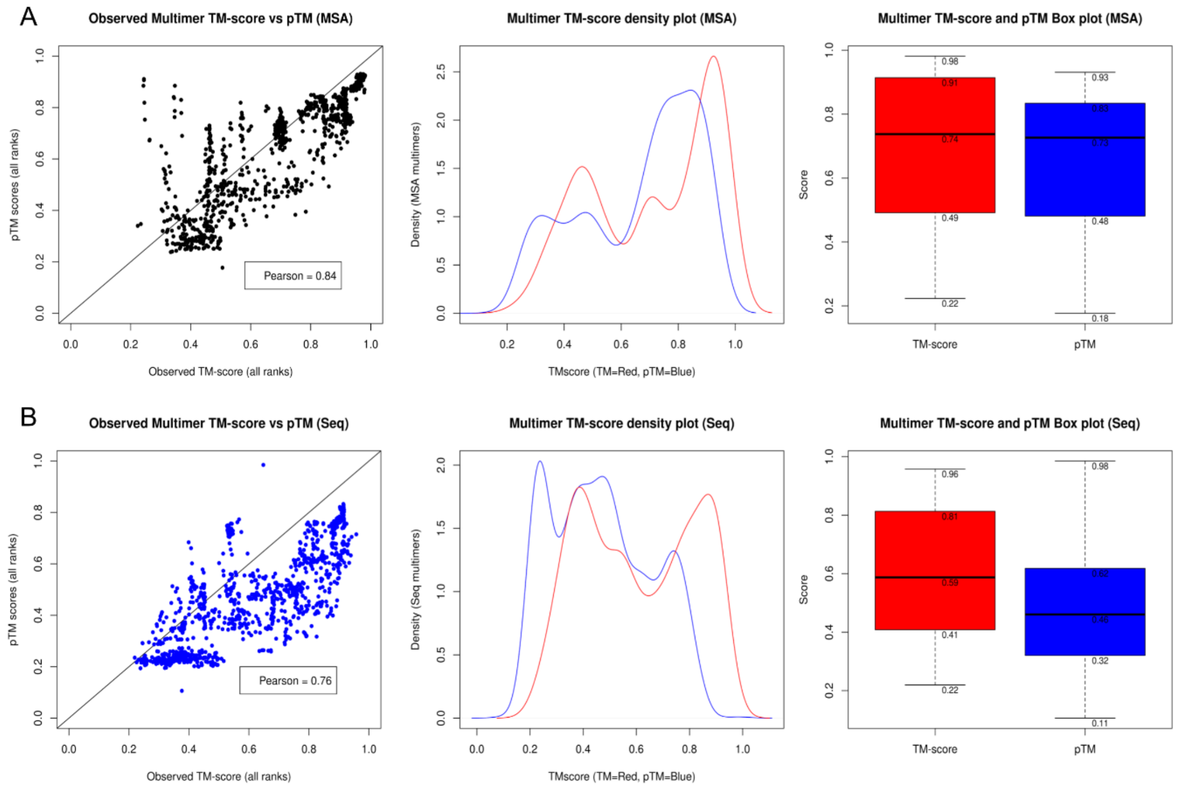

Figure S12. Plots for Population D (Recycled multimers) compared by TM-scores. Scatter plots (left), density plots (middle) and box plots (right). Panel A: plots for MSA recycling and panel B: plots for single sequence (labelled Seq) recycling.

Squared error and MSE were calculated between predicted and observed scores in the same way as described for population C above. The MSE for MSA recycled models was calculated as 0.0174 and for the single sequence models as 0.0380 showing that the single sequence models had a greater overprediction of pTM than the MSA models. The squared error values were again compared using a Wilcoxon rank sum test and this showed that the difference between the values was significant as shown in Table S5 row 1.

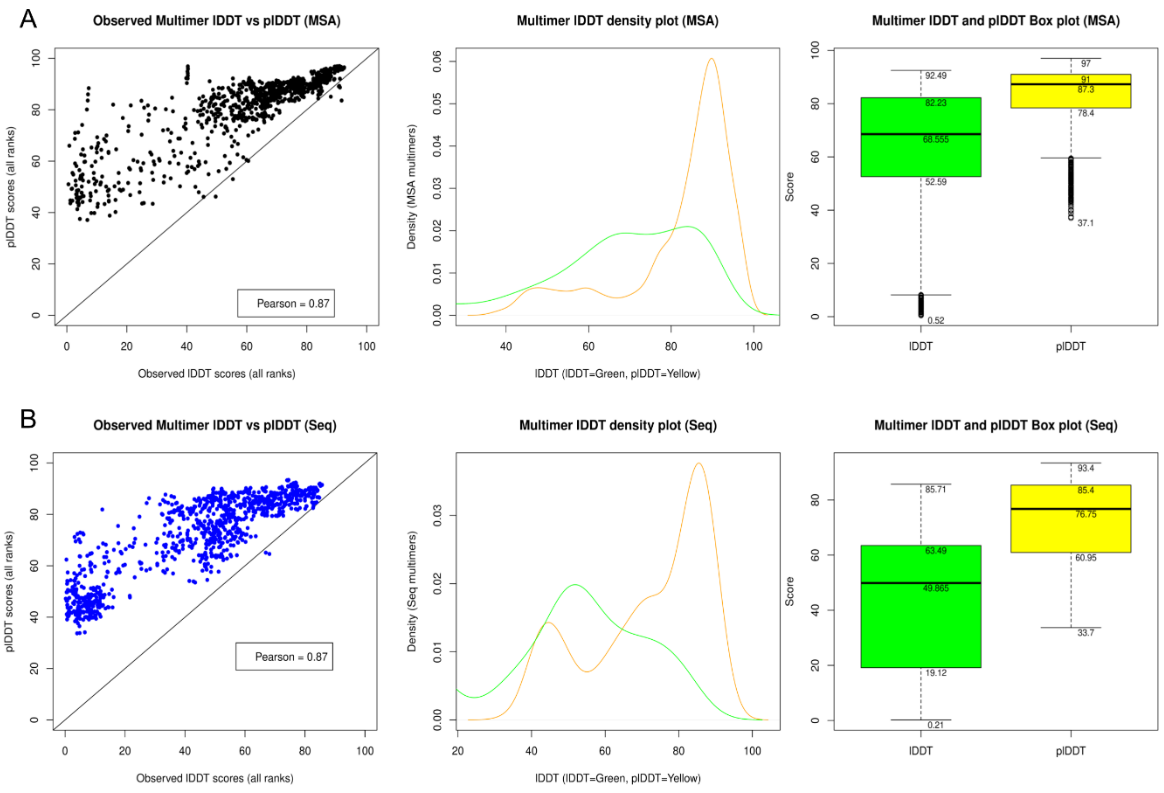

Figure S13. Plots for Population D (Recycled multimers) compared by lDDT scores. Scatter plots (left), density plots (middle) and box plots (right). Panel A: plots for MSA recycling and panel B: plots for single sequence (labelled Seq) recycling.

Similarly for lDDT comparisons, it was found by calculation of squared error and MSE values that models recycled by single sequence had a larger MSE (936.5) than those recycled using an MSA (587.8) and that when comparing the squared error results directly using a Wilcoxon rank sum test (Table S4, row 2) the difference was found to be significant. As the plDDT values had been found to be significantly higher than the oligo-lDDT values for the whole population, this was again tested for both the MSA and single sequence subpopulations. Rows 3 and 4 in Table S5 show that plDDT scores remained significantly higher than oligo-lDDT observed scores as measured by a Wilcoxon signed rank test for both MSA and single sequence subpopulations.

Table S5. Population D. Wilcoxon rank sum test results for the comparison of the squared error values calculated as (pTM – TM-score)^2 between MSA and SS mode (row 1) and as (plDDT – oligo-lDDT)^2 (row 2). Values for a Wilcoxon signed rank test between plDDT and oligo-lDDT within each subpopulation; MSA mode (row 3) and SS mode (row 4) (significant figures in bold).

Row	Scores compared	Independence and distribution symmetry	p-value
1	Squared Errors for MSA and SS groups (TM scores).	Unpaired; 2-sided	**2.20×10^{-16}**
2	Squared Errors for MSA and SS groups (lDDT scores).	Unpaired; 2-sided	**2.20×10^{-16}**
3	plDDT and oligo-lDDT scores (MSA)	Paired; 1-sided test, plDDT > oligo-lDDT	**2.20×10^{-16}**
4	plDDT and oligo-lDDT scores (SS)	Paired; 1-sided test, plDDT > oligo-lDDT	**2.20×10^{-16}**

It appears then, that recycling increases plDDT score variation when using both MSA and single sequence models but that the predicted score variation is greater when an MSA is not performed. Further, that recycling also appears to increase variation in pTM score but that the relationship to MSA or single sequence modes is less clear.
